# Martial Arts “*Kendo*” and the Motivation Network During Attention Processing: An fMRI Study

**DOI:** 10.3389/fnhum.2019.00170

**Published:** 2019-05-22

**Authors:** Hironobu Fujiwara, Tsukasa Ueno, Sayaka Yoshimura, Kei Kobayashi, Takashi Miyagi, Naoya Oishi, Toshiya Murai

**Affiliations:** ^1^Integrated Clinical Education Center, Kyoto University Hospital, Kyoto, Japan; ^2^Department of Neuropsychiatry, Graduate School of Medicine, Kyoto University, Kyoto, Japan; ^3^Artificial Intelligence Ethics and Society Team, RIKEN Center for Advanced Intelligence Project, Tokyo, Japan; ^4^Department of Neurodevelopmental Psychiatry, Habilitation and Rehabilitation, Kyoto University, Kyoto, Japan; ^5^Medical Innovation Center, Kyoto University Graduate School of Medicine, Kyoto, Japan

**Keywords:** *Budo*, *Kendo*, motivation network, functional connectivity, attention

## Abstract

Japanese martial arts, *Budo*, have been reported to improve cognitive function, especially attention. However, the underlying neural mechanisms of the effect of *Budo* on attention processing has not yet been investigated. *Kendo*, a type of fencing using bamboo swords, is one of the most popular forms of *Budo* worldwide. We investigated the difference in functional connectivity (FC) between *Kendo* players (KPs) and non-KPs (NKPs) during an attention-related auditory oddball paradigm and during rest. The analyses focused on the brain network related to “motivation.” Resting-state functional magnetic resonance imaging (rs-fMRI) and task-based fMRI using the oddball paradigm were performed in healthy male volunteers (14 KPs and 11 NKPs). Group differences in FC were tested using CONN-software within the motivation network, which consisted of 22 brain regions defined by a previous response-conflict task-based fMRI study with a reward cue. Daily general physical activities were assessed using the International Physical Activity Questionnaire (IPAQ). We also investigated the impact of major confounders, namely, smoking habits, alcohol consumption, IPAQ score, body mass index (BMI), and reaction time (RT) in the oddball paradigm. Resting-state fMRI revealed that KPs had a significantly lower FC than NKPs between the right nucleus accumbens and right frontal eye field (FEF) within the motivation network. Conversely, KPs exhibited a significantly higher FC than NKPs between the left intraparietal sulcus (IPS) and the left precentral gyrus (PCG) within the network during the auditory oddball paradigm [statistical thresholds, False Discovery Rate (FDR) < 0.05]. These results remained significant after controlling for major covariates. Our results suggest that attenuated motivation network integrity at rest together with enhanced motivation network integrity during attentional demands might underlie the instantaneous concentration abilities of KPs.

## Introduction

Physical exercise is widely believed to be beneficial to health. These benefits are felt by walking, gymnastics, and sports as hobbies. In addition to the physical benefits of exercise on cardiovascular, respiratory, and metabolic systems (Paffenbarger et al., 1984; Biddle et al., 2011), several reports have suggested that engagement in sports positively influences mental well-being. For example, habitual exercise has been found to alleviate depression and anxiety and reduce stress (Hassmén et al., 2000; Callaghan, 2004), and single bouts of exercise have been reported to suppress the urge to drink alcohol (Ussher et al., 2004). Engaging in sport also has beneficial effects on cognitive function (Etnier et al., 1997; Northey et al., 2018), particularly attention. Using a selective attention task, Abernethy and Russell (1987) reported outstanding attentional capacities of athletes. Kida et al. (2005) found that professional baseball players had shorter reaction times (RTs) to target stimuli during a Go/NoGo task. Furthermore, in this study, a 2-year longitudinal follow-up showed a further shortening of RTs, which indicates that practice positively influenced performance (Kida et al., 2005).

The potential benefits of martial arts (known as *Budo* in Japan) for both physical and mental health have received particular attention (Woodward, 2009; Bu et al., 2010; Zheng et al., 2015). Although the various forms of *Budo* are often regarded as sports, one of the characteristics of *Budo* that sets it apart from other sports is its emphasis on the mind and heart, which the Japanese martial arts tradition has partially adopted from concepts of Zen Buddhism. *Budo* emphasizes the importance of a calm, unmoving, and undisturbed mind; these aspects are described as Fudoshin (unmoving mind) or Mu (empty mind; Oosterling, 2011). In contrast to the contemplative meditation of the sitting Zen, *Budo* is regarded as “Zen in action” (Oosterling, 2011), and physical training is an essential component. Previous studies have reported outstanding attentional capacities of *Budo* players (Sanchez-Lopez et al., 2014). Integrative mind-body training, such as meditation and martial arts, is known to enhance performance on attentional tasks (Brefczynski-Lewis et al., 2007; Johnstone and Marí-Beffa, 2018). Furthermore, a positive effect of *Budo* on the improvement of attention-deficit/hyperactive disorder symptoms has been reported (Woodward, 2009). Despite evidence for these positive impacts of *Budo* on attention, the underlying neural mechanism has only been investigated by one study. That study reported a difference between skilled and novice players in event-related potentials during a continuous performance test, which is an index of attention processing, over the frontal and limbic lobes (Sanchez-Lopez et al., 2016).

The mechanisms underlying *Budo*-associated benefits on mental health, including an improvement of cognitive function, is not yet known. Investigating the effect of *Budo* on “motivation” can help to address this question, because cognitive functions, including attention, are considered to be influenced by motivation (Robinson et al., 2012). Thus, in the current study, we focused on the motivation network as a possible neural mechanism that could explain the superior attentional skills of *Budo* players. As mentioned above, a calm and undisturbed mind is required to become a *Budo* expert. Furthermore, as is required in any sport, instantaneous concentration is also essential. According to the Drive Theory (Anselme, 2010), motivation acts as a “drive” that provides an organism with the energy required to trigger, maintain, and direct goal-related behaviors, and with a kind of homeostatic trait. The motivational drive continuously influences on our daily behaviors, that is, instantaneous enhancement of motivation in the face of critical aims/goals, followed by its attenuation after satisfaction. We predicted that this “resting vs. attentionally-driven” state switching/change of motivation can be trained and becomes more efficient through the mind-body training of *Budo*.

Recent neuroimaging studies have suggested that brain functional connectivity (FC) can be used to characterize neural circuits that underpin human cognitive functions, including attention processing assessed by visual oddball paradigm (Li et al., 2016), and health-benefits of non-pathological internet use on motivational function (Fujiwara et al., 2018). Furthermore, FC changes in circumstances such as mental fatigue (after engaging in cognitive tasks for a prolonged period, Li et al., 2016; car driving drowsiness, Harvy et al., 2019) have been reported in EEG studies, in addition to FC abnormalities revealed by functional magnetic resonance imaging (fMRI) in mental illnesses such as schizophrenia (Li et al., 2018).

To test our hypothesis, we focused on *Kendo*, which is a type of fencing with bamboo swords that is practiced by over four million people (International Kendo Federation, 2014). We investigated the difference in FC between *Kendo* players (KPs) and non-*Kendo* players (NKPs) within the motivation network (Kinnison et al., 2012; Fujiwara et al., 2018) during both resting state and an attention-related paradigm. Brain regions within the motivation network have been identified using a motivation-related paradigm (Padmala and Pessoa, 2011). The network consists of 22 regions of interest (ROIs) that are well-synchronized in terms of their activity; identified ROIs include the bilateral intraparietal sulcus (IPS), medial prefrontal cortex (MPFC), frontal eye field (FEF), middle frontal gyrus (MFG), anterior insula (aIns), midbrain (MB), putamen (Put), caudate (Caud), nucleus accumbens (NAcc), left inferior parietal lobule (IPL), right rostral anterior cingulate cortex (rACC), supplementary motor area (SMA), and left precentral gyrus (PCG). In addition to the MB and basal ganglia, which constitute the core of the reward system, these ROIs include several cortical regions that are well-synchronized with the core regions. We hypothesized that KPs have: (1) attenuated FC during resting state; and (2) enhanced FC during tasks with an increased attentional-load.

## Materials and Methods

### Participants

Participants were age-matched KPs (*n* = 15) and NKPs (*n* = 15), who were all healthy men. KPs were defined as individuals who were Dan-grade players (i.e., individuals with a career of *Kendo* for over 10 years) who practiced *Kendo* at least twice a week. Two well-trained psychiatrists confirmed that none of the participants had any psychiatric disorder or severe medical or neurological illness. Estimated intelligence quotients were measured using the Japanese Version of the Adult Reading Test (JART; Matsuoka et al., 2006), and all participants fell within the normal range. After the experimental procedures had been fully explained, all participants provided written informed consent before study participation.

The study was approved by the Ethics Committee of the Kyoto University Graduate School and Faculty of Medicine and was conducted in accordance with the Declaration of Helsinki.

### The Assessment of General Physical Activity and Other Life Habits

#### The International Physical Activity Questionnaire

The International Physical Activity Questionnaire (IPAQ; the 7-item short version, Craig et al., 2003) is a self-rating questionnaire that is used to measure the average amount of physical activity over a week. This questionnaire was developed as a tool for cross-national monitoring of physical activity in adults, and the reliability and validity of the short form of Japanese IPAQ have been confirmed previously (Murase et al., 2002). The indices of the questionnaire are as follows: the average exercise intensity = multiplication of METs and duration of exercise [metabolic equivalents (METs) minutes/day] and those of energy consumption (kcal/day)^1^.

#### Body Mass Index

Body mass index (BMI) was calculated using the following formula: body weight (kg)/ the square of height (m^2^).

#### The Index of Smoking and Alcohol Consumption

Smoking and alcohol consumption may potentially influence attention and motivation/reward system; these habits were therefore assessed using the Fagerström Test for Nicotine Dependence (FTND, Heatherton et al., 1991) and the Alcohol Use Disorder Identification Test (CORE-AUDIT, Babor et al., 1992; Hiro and Shima, 1996).

### MRI Acquisition

The fMRI acquisition started with a 360-s resting-state scan (Rest) using a single-shot gradient-echo echo planar imaging (EPI) pulse sequence on a 3-Tesla MRI unit (Tim-Trio; Siemens, Erlangen, Germany) with a 40-mT/m gradient and a receiver-only 32-channel phased-array head coil. During resting-state data acquisition, we instructed participants to visually concentrate on a fixation cross in the center of the screen and to avoid thinking about anything specific. Next, they received instructions on how to complete the oddball task for 25 s. They then performed the auditory oddball task for 390 s. The task included 30 target trials and 150 non-target trials. Participants heard two different sounds, as follows: 30 pink-noise sounds as target stimuli and 150 pure 400-Hz tones as standard stimuli. Tones were presented in a randomized order. The sounds were generated using Audacity 2.1.1. software^2^. All stimuli were presented using E-prime 2.0 software (Psylab, USA) for 200 ms with a randomized inter stimulus interval (ISI) of 1–3 s in 100 ms units. During the task, participants were instructed to differentiate between target and non-target tones by pressing a button as fast and accurately as possible after target stimulus presentation. The total acquisition time for the fMRI was 775 s. Head movement was minimized within the head coil with the use of foam rubber pads.

Structural MRI data were also acquired using 3-dimensional magnetization-prepared rapid gradient-echo (3D-MPRAGE) sequences. The parameters for the 3D-MPRAGE images were as follows: echo time (TE), 3.4 ms; repetition time (TR), 2000 ms; inversion time, 990 ms; field of view (FOV), 225 × 240 mm; matrix size, 240 × 256; resolution, 0.9375 × 0.9375 × 1.0 mm^3^; and 208 total axial sections without intersection gaps. Parameters for the fMRI were as follows: TE, 30 ms; TR, 2500 ms; flip angle, 80°; FOV, 212 × 212 mm; matrix size, 64 × 64; in-plane spatial resolution, 3.3125 × 3.3125 mm^2^; 40 total axial slices; and slice thickness, 3.2 mm with 0.8-mm gaps in ascending order. A dual-echo gradient-echo dataset for B0-field mapping was also acquired for distortion correction.

### Image Preprocessing

The fMRI dataset was corrected for EPI distortion using FMRIB’s Utility for Geometrically Unwarping EPIs (FUGUE), which is part of the FSL software package (FMRIB’s software library ver. 5.0.9)^3^ and which unwarps the EPI images based on fieldmap data. Artifact components and motion-related fluctuations were then removed from the images using FMRIB’s ICA-based X-noiseifier (FIX; Griffanti et al., 2014).

The preprocessed fMRI and structural MRI data were then processed using the CONN-fMRI FC toolbox (ver.17e)^4^ with the statistical parametric mapping software package SPM12 (Wellcome Trust Centre for Neuroimaging)^5^. First, all functional images were realigned and unwarped, slice-timing corrected, coregistered with structural data, spatially normalized into the standard MNI space (Montreal Neurological Institute, Canada), outlier detected (ART-based scrubbing), and smoothed using a Gaussian kernel with a full-width-at-half maximum (FWHM) of 8 mm. All preprocessing steps were conducted using a default preprocessing pipeline for volume-based analysis (to MNI-space). Structural data were segmented into gray matter, white matter (WM), and cerebrospinal fluid (CSF), and normalized in the same default preprocessing pipeline. Principal components of signals from WM and CSF, as well as translational and rotational movement parameters (with another six parameters representing their first-order temporal derivatives), were removed using covariate regression analysis by CONN. Using the implemented CompCor strategy (Behzadi et al., 2007), the effect of nuisance covariates, including fluctuations in fMRI signals from WM, CSF, and their derivatives, as well as realignment parameter noise, were reduced. As recommended, band-pass filtering was performed with a frequency window of 0.008–0.09 Hz. This preprocessing step was found to increase retest reliability. Before running FIX, movement during fMRI was evaluated using frame-wise displacement, which quantifies head motion between each volume of functional data (Power et al., 2012). Participants were excluded if the number of volumes in which head position was 0.5 mm different from adjacent volumes was more than 20% (Fujiwara et al., 2018). In actuality, no participants were excluded according to this criterion. Furthermore, there was no significant difference in frame-wise displacement between the KPs and NKPs (0.154 ± 0.062 vs. 0.145 ± 0.050, *p* = 0.72).

### Functional Connectivity Analysis

#### The Analysis Within the Motivation Network

We conducted a region of interest (ROI)-to-ROI FC analysis. We specified 22 spherical clusters with 10-mm diameters and peak-coordinates based on motivation-related fMRI studies (Kinnison et al., 2012; Fujiwara et al., 2018). The ROIs were located in the bilateral IPS (IPS_R: *x* = 24, *y* = −54, *z* = 40, IPS_L: −27, −52, 41), MPFC (MPFC_R: 6, 8, 39, MPFC_L: −8, 7, 39), FEF (FEF_R: 34, −11, 48, FEF_L: −31, −12, 50), MFG (MFG_R: 26, 46, 25, MFG_L: −28, 35, 29), aIns (aIns_R: 31, 17, 11, aIns_L: −35, 26, 5), Midbrain (MB_R: 7, −15, −8, MB_L: −10, −18, −8), Put (Put_R: 17, 9, −2, Put_L: −19, 9, 2), Caud (Caud_R: 10, 9, 2, Caud_L: −10, 9, 2), NAcc (NAcc_R: 13, 6, −7, NAcc_L: −13, 6, −7), left IPL (IPL_L: −28, −42, 41), right rACC (rACC_R: 13, 39, 8), right SMA (SMA_R: 0, −6, 57), and PCG (PCG_L: −48, −4, 37). For each subject, the preprocessed fMRI time series of all voxels in the 22 ROIs was extracted and averaged. ROI-to-ROI FC was defined as the Fisher-transformed bivariate correlation coefficients for each pair of the 22 regions, which resulted in a 22 × 22 correlation matrix (231 FCs) in each subject.

Due to the exploratory nature of this study, corrections for multiple comparisons were performed using the False Discovery Rate (FDR), but not using Bonferroni correction (statistical significance *p* < 0.0023) based on the number of ROIs within the motivation network.

#### The Analysis Within the Attention Network

Since we adopted an attention-related paradigm, an analysis within the attention network was conducted to test whether the KP was different from NKP group in attention, referring ventral/dorsal attention network (VAN/DAN; Yeo et al., 2011) as an additional analysis.

### Statistical Analysis

Subject-specific connectivity matrices for each ROI estimated from the CONN toolbox were used as a second-level analysis. We performed a one-way analysis of covariance (ANCOVA) with group (KP vs. NKP) as an independent variable, FC as a dependent variable, and age as a covariate of no interest. Significant connections were identified by calculating the FDR-corrected two-sided *p*-values < 0.05.

A two-tailed *t*-test was applied for group comparisons of demographic data, average RT to the target stimuli in the oddball paradigm, and measures of physical exercise.

To test the effects of smoking and alcohol consumption, general physical exercise, and oddball task RTs on the FC differences between KP and NKP groups, additional analyses were performed in two steps, including a correlational analysis to investigate the association of FTND, CORE-AUDIT, IPAQ, BMI, and RT with FC, and an ANCOVA using group as an independent variable, FC as a dependent variable, and (1) FTND, CORE-AUDIT, (2) IPAQ, BMI, (3) RT, and (4) all covariates of (1), (2) and (3), as covariates. A one-sample Kolmogorov–Smirnov test revealed that the data were mixed in their distribution. Therefore, to test the correlations mentioned above, Pearson’s correlation coefficients were used if an initial exploration of the dataset indicated normal distribution of the data, and Spearman’s rank-correlation coefficients were used if the data were not normally distributed.

## Results

### Demographic Information, General Physical Activity, and Behavioral Data

According to structural MRI findings, one and four subjects were excluded from the KP and NKP group, respectively, because of subtle organic brain abnormalities (ischemic changes or arachnoid cyst). Data from a final total of 14 KPs and 11 NKPs were analyzed. Demographic information, IPAQ scores, BMI, and behavioral data of the oddball paradigm are summarized in Table 1. There were no significant between-group differences in smoking (one person was currently a smoker in each group, and six/five KPs/NKPs were past smokers), alcohol consumption, IPAQ score, or BMI.

**Table 1:** Participant demographics and behavioral data.

	*Kendo* player (*n* = 14)		Non-*Kendo* player (*n* = 11)		
Variables					*p*
Age	39.9	13.0	38.8	13.2	0.836
FTND	0.3	1.1	0.2	0.6	0.776
CORE_AUDIT	7.0	5.0	7.5	7.2	0.824
IPAQ (METs. minutes/day)	439.6	372.3	315.9	202.3	0.336
IPAQ (kcal/day)	483.7	512.7	384.3	268.2	0.584
BMI	23.3	6.4	24.1	3.6	0.708
Auditory oddball paradigm					
Number of omission errors	0.1	0.3	0.0	0.0	0.387
Number of commission errors	0.3	0.6	0.3	0.5	0.285
Reaction time (ms)	171.2	42.9	244.9	91.8	0.028*

FTND, Fagerström test for nicotine dependance; CORE_AUDIT, The alcohol use disorder identification test; IPAQ, International Physical Activity Questionnaire; BMI, body mass index. *p < 0.05.

Regarding behavioral data of the oddball paradigm, the error rate was not significantly different between the two groups. RTs to the stimuli were significantly shorter in KPs than in NKPs (Table 1).

### Group Differences in FC Within the Motivation Network

**Figure 1:**
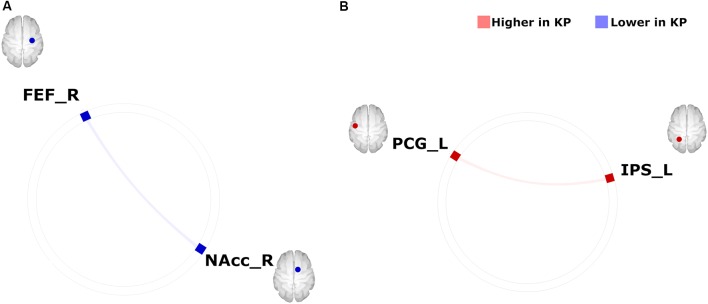
Group differences in FC within the motivation network on **(A)** resting-state-functional magnetic resonance imaging (rs-fMRI) and **(B)** task-based fMRI during an auditory oddball paradigm (KPs < NKPs/ KPs > NKPs). FEF, frontal eye field; FC, functional connectivity; NAcc, nucleus accumbens; PCG, precentral gyrus; IPS, intraparietal sulcus; L, left; R, right; KP, *Kendo* players; NKP, Non-*Kendo* players.

Between-group differences (KPs vs. NKPs) in FC between two regions of the motivation network are shown in Figure 1. The CONN-toolbox analysis revealed the following: (1) KPs exhibited a significantly lower FC between the right NAcc and right FEF (*T*_(22)_ = −4.44, *p* = 0.004) compared with NKPs during rs-fMRI (Figure 1); and (2) KPs had a significantly higher FC between the left PCG and left IPS (*T*_(22)_ = 4.33, *p* = 0.006) than NKPs during the oddball paradigm (all statistical thresholds were FDR < 0.05). No other between-group differences in FC were found.

### Correlations Between FC and Other Variables

The RT to the target stimuli was negatively correlated with FC between the left IPS and left PCG within the motivation network. None of the other variables, that is, FTND, CORE-AUDIT, IPAQ score, and BMI, were correlated with FCs at either resting state or during the oddball task (Table 2).

**Table 2:** Correlations between functional connectivity (FC) and other variables that may be associated with attention.

	FC_FEF_NAcc		FC_IPS_PCG	
	Statistic	*p*	Statistic	*p*
FTND	*ρ* = 0.11	0.609	*ρ* = 0.01	0.972
CORE_AUDIT	*ρ* = 0.01	0.965	*ρ* = −0.28	0.182
IPAQ (METs. minutes/day)	*ρ* = −0.25	0.235	*ρ* = 0.18	0.388
IPAQ (kcal/day)	*r* = −0.21	0.235	*r* = 0.16	0.458
BMI	*ρ* = 0.18	0.402	*ρ* = −0.01	0.955
Reaction time to Odd (ms)	*ρ* = 0.30	0.140	*ρ* = −0.48	0.015*

FTND, Fagerström test for nicotine dependance; CORE_AUDIT, The alcohol use disorder identification test; IPAQ, International Physical Activity Questionnaire; BMI, body mass index; Odd, auditory oddball paradigm; FC_FEF_NAcc, functional connectivity between the frontal eye field and nucleus accumbens; FC_IPS_PCG, functional connectivity between the intraparietal sulcus and precentral gyrus. *Uncorrected p < 0.05.

### Group Comparisons of FC

An ANCOVA was performed using the following variables as covariates: (1) FTND and CORE-AUDIT as life habits that affect attention and motivation; (2) IPAQ score and BMI as indicators of general physical activity; (3) RT; and (4) all covariates of (1), (2) and (3), as a factor that has a significant effect on the between-group differences in FC (Table 3). The ANCOVA revealed that, compared with the NKP group, KPs had a significantly lower FC between the right FEF and right NAcc during rest and higher FC between the left IPS and left PCG during the oddball task after controlling for covariates (1), (2), (3) and (4; Table 3). According to the ANCOVA analyses, there were no significant effects of covariates (FTND scores, CORE_AUDIT, IPAQ score, BMI, and RTs) on the differences of FC.

**Table 3:** Group comparison of FC by analysis of covariance (ANCOVA) controlling for confounding factors.

	Group effect of FC_FEF_NAcc		Group effect of FC_IPS_PCG	
Covariates	Statistic	*p*	Statistic	*p*
FTND, CORE_AUDIT	*F*_(1,21)_ = 6.927	0.002	*F*_(1,21)_ = 6.850	0.002
IPAQ (MET-minutes/week), IPAQ (kcal/day), BMI	*F*_(1,20)_ = 4.909	0.008	*F*_(1,20)_ = 3.427	0.031
Reaction time to Odd (ms)	*F*_(1,22)_ = 9.998	0.001	*F*_(1,23)_ = 9.592	0.001
FTND, CORE_AUDIT, IPAQ(MET-minutes/week), IPAQ (kcal/day),	*F*_(1,16)_ = 11.971	0.003	*F*_(1,16)_ = 7.146	0.017
BMI and Reaction time to Odd (ms)				

*FTND, Fagerström test for nicotine dependance; CORE_AUDIT, The alcohol use disorder identification test; IPAQ, International Physical Activity Questionnaire; BMI, body mass index; Odd, auditory oddball paradigm; FC_FEF_NAcc, functional connectivity between the frontal eye field and nucleus accumbens; FC_IPS_PCG, functional connectivity between the intraparietal sulcus and precentral gyrus*.

### Comparison of FC Within the Attention Network

No differences were found between the KP and NKP groups in FC during either resting state or the oddball paradigms in the analysis within the VAN/DAN.

## Discussion

In the current study, we predicted that “resting vs. attentionally-driven” switching/change of motivation can be trained and becomes more efficient through the mind-body training of *Budo*. The main finding of this study was that one of the FCs (FEF-NAcc) within the motivation network was smaller during rest in KPs vs. NKPs, while one of the FCs (PCG-IPS) was larger during the higher attentional load required during the oddball task in KPs vs. NKPs. This result is in line with our initial hypothesis and indicates that KPs can recruit the motivation network in a more timely manner, depending on the attentional demand.

This is the first study to investigate the neural correlates of the effect of *Kendo* on motivation, focusing on FC within the motivation network during both resting state and an attention-related task. We also investigated the effects of confounding factors on between-group differences in FC, including smoking, alcohol consumption, and general physical activity. As a result, no effect of the potential confounding factors on the difference in FCs was found.

*Budo* has elements of both Zen and actual physical training. The emphasis on having a calm/empty mind, much like Zen, distinguishes *Budo* from other sports. This Zen spirit is substantiated in the training style of *Budo*. For example, in the case of *Kendo*, a regular training starts and concludes with a short period of meditation called “mokuso,” during which individuals sit silently with their eyes closed (Labbate, 2011). Sport, in general, is likely to have positive effects on attentional processing. This might be due to increased recruitment of the motivation network when motivational drive is needed in response to attentional loads. In the case of *Budo*, and particularly with regard to its element of Zen, decreased recruitment of the motivation network during rest might represent a kind of preparation stage for efficient attentional processing, which could represent a “resting vs. attentionally-driven” contrast in terms of the integrity of the motivation network. This contrast between two states, that is, a resting vs. attentionally-driven state in motivation is consistent with a key concept of Zen, Fudoshin (unmoved mind), which was conceived by Takuan Soho, a Zen master priest (1573–1645). According to his writing “*The Unfettered Mind: Writings from a Zen Master to a Master Swordsman*” (Wilson and Takuan, 2012), an “unmoved mind” is a calm mental state, but with the potential to flexibly move at a moment’s notice; these ideas have been interpreted in different ways. This resting vs. attentionally-driven contrast might explain another key concept of Zen, “Shinshin-ichinyo” (mind-body unity; Nakao and Ohara, 2014). An integrated mind-body training (which includes aspects of Zen, as well as those of physical exercise) by *Kendo* practice could lead to the development of the mind/body in a unified manner.

Only RTs of the oddball paradigm were correlated with FCs, during both rs-fMRI and task-based fMRI. These results suggest that smoking, alcohol, and exercise habits are not associated with FC differences between KPs and NKPs. RTs were shorter in the KP group than the NKP group and were negatively correlated with FC differences between the PCG and IPS during the oddball paradigm. This suggests that faster RTs indicate a stronger neural network integrity between the PCG and IPS. The between-group differences in FC were still significant after controlling for these potential confounding variables. The lack of any between-group difference in attention network integrity might be due to the simple structure of the oddball paradigm and the low cognitive demands in attention processing while performing this very basic task. In this sense, significant between-group differences in FC of the motivation network but not of the attention network in the current study would indicate a difference between KPs and NKPs in cognitive rewarding/motivational drive rather than a difference in attention processing at low cognitive demands.

Although the current results support the idea that *Kendo* brings health benefits, in the sense that strengthens the motivation network favorably, physical exercise, in general, might have negative impacts on mental health when training is excessive, such as “overtraining syndrome” (Kreher and Schwartz, 2012) or exercise dependance/sports addiction (Hausenblas and Downs, 2002). Given this potential effect on mental health, further investigations should be carried out to determine the appropriate quantity, quality, frequency, and intensity of *Kendo* training.

The current study has several technical limitations that should be considered. First, all participants were all male. Therefore, the results of the study cannot be generalized to women. Second, the sample size was relatively small. This could have resulted in a higher risk of type II errors, and so the results should be interpreted with caution. Future work could replicate the current study with a larger sample size. Third, we adopted a cross-sectional design. Considering the potential disadvantages of analyzing data from one time point, it is still unknown whether *Kendo* practice changes FCs in the motivation network or whether KPs have an innate trait of high FC in this network. A longitudinal follow-up study will be necessary to clarify the causal relationship between *Kendo* practice and FC changes within the motivation network and to clarify the specificity of *Kendo* effects on FC, while also considering the effects of other potential confounding variables not included in the present study. Fourth, due to the nature of exploratory studies, Bonferroni correction was not applied in FC analysis for multiple comparisons. However, this could have resulted in type 1 errors. Fifth, we did not examine the differences between any other sports or martial arts. Further studies are needed to clarify whether *Budo*/*Kendo* has specific effects or whether the results can be generalized to other fitness practices.

Finally, in addition to the above-mentioned limitations, the lack of behavioral correlates of brain parameters complicates the interpretation of our results. FC between the left IPS and left PDG during the oddball paradigm was negatively correlated with RTs, which might suggest that higher connectivity in the motivation network is advantageous in realizing higher attentional performance. However, the functional significance of the attenuated connectivity at rest in KPs is unclear, as no behavioral correlates were observed; nonetheless, our interpretation is that reduced connectivity at rest might help us attain favorable psychological states such as serenity. Follow-up studies that include relevant psychological or cognitive measures will be necessary to investigate this. In addition, physiological measures of the autonomic nervous system, such as heart rate and its variability, might also be useful.

In conclusion, we examined the effects of *Kendo* during rest and the oddball paradigm, focusing on the connectivity of the motivation network. We found a lower FC in rs-fMRI and a higher FC during attention-related paradigms. Our results suggest that the contrast between lower activities within the motivation network at resting state and the enhanced ones during the attentional task in KPs are indicative of a difference between KPs and NKPs in terms of motivational drive in attention processing. However, the results should be regarded as preliminary in light of the limitations mentioned above. Further studies with larger sample and a longitudinal study design are needed to verify the present findings. The integrated training of both mind and body, which is substantiated in *Budo*, including *Kendo*, might be applicable to a wide range of health-promoting programs for enhancing cognition and could also inform therapeutic programs for various psychiatric conditions, such as attention-deficit/hyperactive disorder.

## Ethics Statement

The study was approved by the Ethics Committee of the Kyoto University Graduate School and Faculty of Medicine and was conducted in accordance with the Declaration of Helsinki.

## Author Contributions

HF conceived, designed, and conducted the experiments, acquired and analyzed the data, and drafted the manuscript. TMu, TU, and HF contributed to the conception of the study, interpretation of data, and revisions for critically important intellectual content. KK, SY, TMi, and NO contributed to the design and data acquisition, interpretation of data, and drafting the manuscript. All authors approved the final manuscript for submission and agree to be accountable for all aspects of the work, including the assurance that questions related to the accuracy or integrity of any part are appropriately investigated and resolved.

## Conflict of Interest Statement

The authors declare that the research was conducted in the absence of any commercial or financial relationships that could be construed as a potential conflict of interest.

## Footnotes

^1^https://sites.google.com/site/theipaq/scoring-protocol

^2^https://www.audacityteam.org/

^3^http://www.fmrib.ox.ac.uk/fsl

^4^www.nitrc.org/projects/conn

^5^http://www.fil.ion.ucl.ac.uk/spm
